# The Schema and Organization of the Cell: An Introduction to Ernst Brücke’s *Die Elementarorganismen* (1861)

**DOI:** 10.1007/s10739-024-09774-8

**Published:** 2024-08-16

**Authors:** Daniel Liu

**Affiliations:** Historisches Seminar, Ludwig-Maximilians-Universität München, Geschwister-Scholl-Platz 1, 80539 Munich, Germany

**Keywords:** Cell, Protoplasm, Organism, Organization, Physiology, Histology, Microscopy, Muscle, Connective tissue, Intestinal epithelium

## Abstract

Ernst Brücke’s 1861 essay *Die Elementarorganismen* has often been cited as a watershed in the history of physiology as well as in the history of cell theory. In its time it was widely read as a reform of animal cell theory, shifting the concept of the cell away from Schleiden and Schwann’s original cell schema of a membranous vesicle with a nucleus, and towards the protoplasm theory that had developed in botany, centered on the cell’s living contents. It was also notorious for its arguments against the necessity of both the nucleus and the cell membrane. An English translation of “The Elementary Organisms” is presented for the first time in this journal issue, with annotations and illustrations, https://doi.org/10.1007/s10739-024-09773-9. Brücke’s essay was not only an intervention into cell theory: historians can read it as a continuation of debates on the nature of the organism and theories of organization, and as an epistemological meditation on the microscope. In addition, although Brücke was known as a founder of the Berlin school of organic physics, “The Elementary Organisms” shows how he combined an avant-garde physicalist physiology with a much older tradition of comparative anatomy and physiology. The following introductory essay will provide a scientific biography of Ernst Brücke up to 1863, with background on debates on biological organization, cell theory, and muscle histology.

On October 10, 1861, the physiologist Ernst Brücke submitted an essay titled “The Elementary Organisms” to the Imperial Academy of Sciences in Vienna, which is translated into English here for the first time (Brücke 2024). Contemporaries saw Brücke’s essay as a major inflection point in the development of cell theory: it was a clarion call to reform the 1838/39 cell theory of Matthias Schleiden and Theodor Schwann, an exhortation to search for the inner structure of the cell, and a reminder that cell theory’s value was as a physiological theory as well as a morphological one. Historians have regarded it as an important precursor to Claude Bernard’s theory of the *milieu intérieur* (Holmes 1963), as a starting point for particulate theories of heredity (Churchill 2015, p. 390; Bolduc 2021, pp. 195–198), and the origin of the idea that cells as “elementary organisms” are irreducible units of life (Schloegel and Schmidgen 2002; Dröscher 2002; Reynolds 2008; Dröscher 2016; Reynolds 2018). Brücke was cited by his contemporaries and later by historians as a promoter of protoplasm theory—that, in Brücke’s words, protoplasm was “the living cell body” (*der lebendige Zellenleib*)*.* It was famous for its argument that neither the membrane nor the nucleus is essential to the definition of the cell itself, a radical claim that led to decades of debate about the correct way to define the cell (Dröscher 2002; Liu 2019).

Yet Brücke’s 1861 essay is not so easy to summarize. It was a survey of recent research; a look at the many exceptions to cell theory’s original rules that were laid down by Schwann in 1838/39; it was a paper by a teacher who wanted to promote work done by his students; and it remains a durable reflection on the nature of microscopic vision. But most of it, by quantity at least, was an attack on the prevailing definition or “schema” of the cell as having a “solid cell membrane, initially fluid cellular content, and cell nucleus with nucleoli.” Reynolds (2010) has reminded historians of biology that, although cell theory has been under constant revision since the 1830s, it has remained an enduring biological law with many uses across fields of study (see also Richmond 2000). “The Elementary Organisms” ranks among the most aggressive attempts to reform cell theory. Arriving a little more than two decades after Schleiden and Schwann’s 1838/39 cell theories, Brücke’s essay contained hints of a more modern conception of the cell, one focused on its internal structure and dynamics. It also predates the discovery of the chromosomes and mitosis by nearly two decades, and Brücke’s attacks on the necessity of the cell membrane and nucleus betray how immature cytology was in 1861. Brücke’s “Elementary Organisms” was also not the most important call for reform to cell theory in 1861. That distinction belongs to a paper by the anatomist Max Schultze (1825–1874), whose straightforward redefinition of the cell as a “glob of protoplasm with a nucleus” (Schultze 1861, p. 11) both preceded Brücke’s essay by a few months, and was also more widely cited (e.g., in Haeckel 1862; Strasburger 1876; Flemming 1882). Nevertheless, Brücke’s essay is worth revisiting by historians not only as a commentary on cell theory, but because its sweep touched on so many different areas of biological research in the mid-19th century. In particular, “The Elementary Organisms” provides historians with an excellent view into theories of the organism and biological organization, as articulated by a staunch physicalist physiologist at the tail end of the German debate over scientific materialism (Gregory 1977; Bayertz et al. 2012). The following short introduction will provide background to Ernst Brücke the scientist, his ideas about organization and the cell, and the immediate circumstances of the essay’s composition in 1861.

Ernst Wilhelm Brücke (1819–1892) was born in Berlin and raised in the city of Stralsund by his mother’s sister Johanna and her husband Friedrich Bernhard Droysen (1761–1838), superintendent of the Stralsund parish and pastor of the city’s famous St. Nicholas Church. As a youth, Brücke wanted to become a painter like his father, but thought himself not talented enough. He then entertained the idea of becoming a farmer or a shipwright, but shipbuilding in Stralsund had stagnated, with the harbor still filled with rubble dumped there by Napoleon’s retreating soldiers, and the shipwrights’ guild unable to make the shift from sail to steam (Ewe 1985). Brücke instead decided to become a physician, and began his studies at the University of Berlin in October 1838—mere months after Matthias Schleiden had published his theory of plant cells in “Contributions to Phytogenesis,” and in the midst of Schwann’s furious writing of his cell theory in his *Microscopic Investigations* (Schwann 1847, p. *xviii*; Watermann 1960, p. 97). There is no indication that Brücke met the two founders of cell theory when they were still in Berlin: Schleiden left Berlin amidst a personal scandal in December 1838 (Jahn and Schmidt 2005), and Schwann departed for a professorship in Louvain the following April amidst a crisis of faith (Watermann 1960, p. 24; Vienne 2017). Brücke began studying directly under Schwann’s teacher Johannes Müller (1801–1858) in 1841, and became Müller’s assistant at the university’s museum for comparative and pathological anatomy in 1843—the same position Schwann had held in the 1830s. Brücke would have been surrounded by talk of Schwann’s cell theory and microscopic anatomy in general, and not just from Müller’s vigorous promotion of Schwann’s theory. Berlin was a hotspot for microscopy in anatomy, botany, protistology, and mineralogy, and was also home to Friedrich Wilhelm Schiek (1790–1870), one of the best microscope makers of the 1830s and 1840s (Jahn 1987; Dierig 2001; Otis 2007; Gerlach 2009, pp. 262–264).

Cell theory and histology were only means to greater ends for Brücke: physiology, and a physicalist perspective on life. Brücke cultivated this perspective in good company. In early 1841 he met another of Müller’s students, Emil du Bois-Reymond (1818–1896). The two became lifelong friends, sharing worldviews, politics, and scientific commitments:

Brücke and I have sworn to assert the truth that no forces operate in the organism other than the common physical-chemical ones; that, where these do not yet provide sufficient explanation by the physical-mathematical method, either the type or manner of their effects must be sought in a definite case, or that new forces must be assumed that are inherent in matter, are of the same dignity as physical-chemical [forces], and can always be traced back to repulsive or attractive components.^1^

In 1845 du Bois-Reymond and Brücke were two of the founding members of the Berlin Physical Society, a group of upstart young scientists with passions for physics, instruments, and engineering (Wise 2018). Werner Siemens joined in 1845, and both Hermann Helmholtz and Carl Ludwig joined the group in 1847, a moment that the classical historiography has called the beginning of “organic physics” (Cranefield 1957, 1966a; Lenoir 1988). The group grew to sixty-one members before 1848, and met to share papers, lectures, and discussions; many of Brücke’s articles from this period mention they were first presented at the Berlin Physical Society. But there was diversity within this ideological community, and Brücke’s version of this physicalist perspective was neither as radical nor as speculative as some. For example, du Bois-Reymond felt free to speculate about molecular models of muscle to explain its electrophysiology (Finkelstein 2013, chap. 4), whereas in “The Elementary Organisms” Brücke showed a little more positivistic restraint and was unwilling to make such a molecular leap about cellular structure. Nor was Brücke an experimentalist like du Bois-Reymond, Ludwig, or Helmholtz were.

Instead, Brücke’s particular skill was in his ability to compare histological structures to simplified physical models, as well as to compare the physical properties of one histological structure across species. In the 1840s, for example, Brücke studied the rod cells of the retina as if they were prisms of colored glass (Brücke 1844), and modeled the geometrical optics of each part of the eye by constructing “semiartificial” eyes out of eyes of different animals (Brücke 1845a; Schickore 2007, p. 211). His position at Müller’s anatomy museum gave him the materials to extend his study of retinal reflection across vertebrate orders from fish to cetaceans (Brücke 1845b). When Brücke received a live chameleon as a gift, he took the opportunity to compare its color changing skin to that of various cephalopods, showing how light diffracting cells interacted with pigmented cells (Brücke 1852a, b). The novelty of these studies was that they combined physical reasoning (e.g., wave theory, geometrical optics) with simple histology done extensively across species. As Schickore (2000, 2007) has shown in her work on the history of vision theory, Brücke’s work on the eye demonstrated how light moved through its parts, refracting and reflecting. But Brücke never arrived at a physiological theory of vision. Other anatomists with finer, more intensive histological skills were able to show that the rods and cones of the retina were not just refractive prisms, but were themselves nerve cells, and therefore the actual light-sensing organs. Brücke seems to have possessed keen insight into the physical properties of cells, as well as experience dissecting many animal species, but he possessed only middling skill in histological microtechnique. He could even be dismissive of “the closed phalanx of pure morphologists and chaste observers” and the “pure, immaculate microscopist[s]” (Lesky 1976, p. 235).^2^

Brücke’s physiological interests were more also diverse than du Bois-Reymond’s, who stuck to a single research area for his whole life. Brücke’s breadth would inform the arguments he would make in “The Elementary Organisms,” and they also turned out to be a significant career advantage. His experience preparing specimens for Müller won him a lectureship in anatomy in 1846 at the Royal Prussian Academy of Art, and his work on the anatomy and physiology of the eye won him a further promotion as Müller’s prosector in 1847 (Schnitzler 1890, p. 1177; E. T. von Brücke 1928, p. 25). Brücke had held that job for only a few months when he received the call to replace Karl Friedrich Burdach as professor of physiology in Königsberg (now Kaliningrad). He departed from Berlin at the end of March 1848, stopping first in Stralsund to marry his childhood friend Dorette Brünslow (1819–1893). His timing was fortunate. Brücke had witnessed some of the worst violence of the 1848 Revolutions in Prussia just weeks before he left Berlin, but in Königsberg he was politically and geographically insulated.^3^ In December 1848 Brücke was recruited to the University of Vienna by the anatomist Josef Hyrtl (1810–1894). The medical faculty in Vienna had been in turmoil in 1848, culminating in the dismissal of five professors, including the former professor of physiology, as well as the minister of education (Lesky 1976, chap. III.1). In this chaotic vacuum a lack of political credentials was obligatory if Brücke was to be hired and survive in Vienna: the conservative university was already reluctant to allow a Prussian Protestant like Brücke to become a professor, and Hyrtl had to work to circumvent the Austrian law barring foreigners from holding university posts.^4^ But the need to improve university teaching was urgent, and educational reform would become one of the handful of durable reforms of the Revolutions of 1848 in Austria (Lesky 1976; Coen 2007; Klemun 2016). The Viennese reformers also recruited the botanist Franz Unger (1800–1870), whose expertise in the anatomy and physiology of plants was a mirror to Brücke’s expertise in animals (Klemun 2016). In the reactionary years after 1848 the stakes for these reformers could be quite high: in Unger’s case, his appointment drew attacks from conservative Catholics for importing “German materialism” (e.g., evolutionism) into Austria, despite the fact that Unger was himself Catholic, Austrian, and not much of a materialist (Gliboff 1998).

Among Brücke and Unger’s remits in Vienna were to train students in microscopy, and Brücke has been said to have taught microscopy to “thousands” of medical students (Lesky 1976, p. 465; Dröscher 2016). Indeed, one can read “The Elementary Organisms” as promoting or defending his students’ research on the structure of epithelial cells, muscle fibers, and cartilage. Two of Brücke’s assistants, Salomon Stricker and Sigmund Exner, would go on to write textbooks on histological and microscopic technique, and Exner’s textbook likely deviated but little from Brücke’s own teaching (Stricker 1870; Exner 1873). Brücke and his students had an idiosyncratic conception of microscopic vision, one that was based on Brücke’s physicalist approach to the physiology of the eye. For the Brückean school, the microscope was not merely an instrument that allows the microscopist to see small things, enlarged and in greater detail. Rather, Brücke understood the microscope as an optical device that projects an image onto the microscopist’s retina (Stricker 1870, p. *i*; Exner 1873, p. 7). As a consequence, understanding what can be perceived with the microscope would require understanding the nature of the light that constitutes the image.

## Brücke on the History of Cell Theory and the Cell’s Invisible Organization

Brücke had two central arguments in “The Elementary Organisms,” one building on the other. His first argument was that the internal contents of the cell “must” be organized and have structure because the cell is alive, and *vice versa*. This claim about the equivalence of life with material organization was part of a long tradition of biological thought that Brücke inherited from Johannes Müller (Rheinberger 1987, among many others), and in what follows I will try to give a simplified genealogy of this tradition. Brücke’s second argument followed from the first: that biologists had misled themselves into believing that the interior of the cell was unorganized, because they did not understand *why* the cell’s contents looked like a transparent fluid under the microscope, and not an organized body.

Organization and the organism are complicated topics in the historiography of biology, and philosophers have long enjoyed playing with the dualisms that are the organism concept’s abiding leitmotifs: epigenesis vs. preformation, and vitalism vs. mechanism (Jacob 1973; Roe 1981; Rheinberger 2000; Huneman and Wolfe 2010; Riskin 2016; Nicholson and Dupré 2018; Zammito 2018; Bognon-Küss and Wolfe 2019; Lyons 2020; Morange 2021). Thankfully, Brücke’s presentation of organization in “The Elementary Organisms” was simpler than most, eschewing any discussion of the laws and causes of the organism’s development. Brücke instead followed another long tradition: drawing a conceptual line, placing life and organized matter on one side of that line, and non-life and unorganized matter on the other. Although Brücke sought to overturn much of Schwann’s cell theory, his approach to life and organization followed directly from Schwann and their teacher Müller. “The Elementary Organisms” shows that the topic of organization persisted even as biology and physiology became more reductionist, physicalist, and mechanistic.

Xavier Bichat’s (1771–1802) *Anatomie générale* of 1801 is not customarily cited in histories of the origins of biology, but Kai Torsten Kanz (2007) has pointed to Bichat’s work as exemplifying the impulse to classify the world into living and non-living beings (see also Foucault 1966; Huneman 1998; Rheinberger 2000; Toepfer 2011, pp. 754–842; Duchesneau 2016). The original text in French and its first English translation are very close, and I have highlighted the relevant terms:

There are in nature two classes of beings, two classes of properties, and two classes of sciences. The beings are either **organic** or **inorganic**, the properties **vital** or **non-vital**, and the sciences **physiological** or **physical**. Animals and vegetables are organic—minerals are inorganic. Sensibility and contractility are vital properties; gravity, elasticity, affinity, &c. are non-vital properties. Animal and vegetable physiology, and medicine form the physiological sciences; astronomy, physics, chemistry, &c. are the physical sciences. (Bichat 1822, p. 9)

Today, our sensibilities about what is “organic” and “inorganic” come from chemistry, where the distinction is based on a static system of classification: it does not make sense to us to think of something making a transition from the inorganic to the organic. The end of the 18th century and beginning of the 19th saw a flourishing of theories of both epigenesis and spontaneous generation, both of which posit the possibility of just such a transition across a sharp conceptual divide (Farley 1977; Roe 1981). Perhaps it is a little less surprising then, that when the physician Christoph Heinrich Pfaff translated Bichat into German in 1802, he easily substituted “organic” with “organized:”

Es giebt in der Natur zwey Klassen von Wesen, zwey Klassen von Eigenschaften, zwey Klassen von Wissenschaften. Die Wesen sind **organisirt**, oder **unorganisirt**, die Eigenschaften **vitale** oder **nicht vitale**, die Wissenschaften **physiologische** oder **physische**. Die Thiere und Pflanzen sind organisirt. Was man Mineralien nennt ist unorganisiert. (Bichat 1802, p. 1)

This bifurcated conception of nature not only persisted into the 1830s, but in some ways became even stronger as physiology, anatomy, and chemistry developed in separate directions. For example, Müller did not believe that organic chemistry would ever explain organismal form, and that therefore only a vital force could explain how organisms could arise from organic matter. “The words organic and organized have invariable physiological definitions for us,” Müller wrote in 1835*.* “For us, organic substances are all of those that are produced by organisms. What is organized are only those parts of the organism which do not just have organic compounds, but have the required organization within themselves for their independent nutrition and growth, *i.e.*, vessels” (Müller 1835, p. *vi*).^5^ For our purposes here it is only important to remember that, in the first half of the 19th century, life was identified with organization and thus organisms. Beyond that, we can understand why the stakes of the epigenesis vs. preformation and vitalism vs. mechanism debates were so high: these were debates about how it was possible to cross the line from unorganized, non-living matter, to organized, living organism. Development and (spontaneous) generation were problems that seemed to provoke radical solutions precisely because the transition from unorganized matter to organized life form was so dramatic.

By the 1830s, however, the nature of the causes of organization had been black boxed, explained by either vague gestures towards some vital force whose existence was asserted but never directly investigated, or by mechanical hypotheses that were even more speculative than the vitalist ones. Müller and his students instead stuck to describing development rather than seeking its causes. These descriptions tended to posit a structureless, fluid substance that coagulated to form developing tissues and organs, although the name and nature of these germinative substances remained ambiguous (Lorch 1967; Pickstone 1973; Parnes 2000; Liu 2017). In 1830 Müller introduced a new term, *blastema*—from the Greek *βλαστάνω*, to sprout or grow, or the noun form *βλάστημα* for foliage or offspring, and also a Hippocratic term for pimples or boils—as a synonym for the German word *Keimstoff*, the “germinative material.” Müller gave no explanation for the new term, describing it only as “*materiem gelatinosam primigeneam, quam blastema liceat postea nominare*” (“this primordial gelatinous substance, which we may later name blastema;” Müller 1830, p. 60). One could imagine that the Teutonic *Keimstoff* just did not look right in a treatise written in Latin. The terms for the germinative fluid had never been precise, and Müller used the word “blastema” loosely as well: for example, when he described the embryonic development of glands, he wrote, “we see the blastema in the form of a gelatinous semi-transparent matter, in which the ramification of the secreting tubes commences in an arborescent form” (Müller 1838, p. 432).

When Theodor Schwann wrote his cell theory in *Microscopic Investigations* in 1838/39, he followed Müller by describing cell genesis as a transition from a “structureless” (*strukturlos*) fluid to the cell, using terms not far removed from Müller’s descriptions of tissue formation. Schwann coined the infamous term *cytoblastema*—but a minor modification of Müller’s blastema—writing: “The generation of the cells takes place in a fluid, or in a structureless substance[…] We will name this substance in which the cells are formed, cell-germinating material (Zellenkeimstoff) or cytoblastema” (Schwann 1847, p. 40). As Müller had with blastema, Schwann did not specify what the cytoblastema was made of, and he was so imprecise about its location that in subsequent decades there was considerable debate over whether the cytoblastema (and thus cell genesis) was found either inside or outside of existing cells, or both. Schwann laid out his theory for cell genesis as a cascading sequence, the so-called theory of “free cell formation.” First a tiny granule, the nucleolus, coagulates in the cytoblastema. The granular nucleolus then develops a surrounding membrane, forming a vesicular nucleus. The nucleus recapitulates this process on a larger scale, converting the surrounding cytoblastema into the cell membrane, then the now-membranous vesicle continues to grow by assimilating more cytoblastema, until “the external stratum becomes more perfectly developed to form a cell” (Schwann 1847, p. 180).

From 1838 and through the 1850s the animal cell was recognizable anatomically as a membrane-bound vesicle (*Bläschen*) that contained a nucleus, which itself was a vesicle containing one or more granular nucleoli. The cell represented a threshold of organization. What Brücke would later call this “schema of [the cell as] a fluid-filled vesicle with a nucleus and nucleolus” was distinct from its unorganized component parts: free-floating nuclear granules and unnucleated vesicles (Kölliker 1853, pp. 11–14). A membrane-bound vesicle by itself was not organized or alive, nor was a free-floating nucleus. Schwann also described the cell membrane and nucleus as themselves structureless (Schwann 1847, pp. 40–43, 56, 138–39, 146, 153, 173), insisting that the cell as a whole was “more perfectly developed” and its process of organization was “more intense and complete” than could be found in a vesicular membrane or nucleus alone (Schwann 1847, p. 180).

But this was as far as Schwann was willing to go (Lenoir 1983, pp. 124–134). Biographers of Schwann have all noted that, in 1838, Schwann was at odds with Müller’s vitalism and his ideas about the ultimate source of the organism’s organization (Watermann 1960; Otis 2007; Vienne 2017).^6^ Schwann also departed from Schleiden, who had famously called the cell a “little organism” that lived its own life: Schwann was comfortable in calling the whole animal organized, but he regarded cells as merely parts of that organization. Thus, his preferred terms were *Elementartheile* (elementary part, used 94 times), *Elementarzelle* (elementary cell, 27 times), and *Elementargebilde* (elementary form or formation, 22 times). Nevertheless, Schwann’s cell theory and his concept of the cell—a membrane bound vesicle containing a nucleus—were understood as having “paved the way to a scientific understanding of the distinction between organic and inorganic forms” (Kölliker 1845, p. 49).

In botany between 1838 and 1861 many reforms were made to Schleiden’s cell theory: botanists developed robust theories of cell development and multiplication, many of which were based on the identification of protoplasm as the living part of the plant cell (Farley 1982; Liu 2017). In animal and medical anatomy these changes were fewer and farther in between. In 1850–1855 Robert Remak and Rudolf Virchow—both students of Müller (see Otis 2007)—replaced Schwann’s theory of free cell formation with the theory of cell division, borrowing from botanists’ theories of cell division from 1846 (Harris 1999, chap. 13).^7^ But the schema of the cell had remained the same, with a membrane, nucleus, and structureless cell contents. In the 1859 edition of Albert Kölliker’s authoritative *Manual of Human Histology*, the cell contents (*Zelleninhalt*) were nothing more than a fluid with other objects floating in it, while the cell was still the “elementary part” of the organism, despite the fact that Kölliker recognized it as possessing its own life (Kölliker 1859, p. 27*ff.*).

Brücke’s argument was thus implicit in the title of his paper: if the cell was to possess its own life, then it must have organization. It is striking, however, that “The Elementary Organisms” made most of its case for the cell’s organization in negative terms. By word count, at least, nearly half of “The Elementary Organisms” was devoted to Brücke’s arguments against the necessity of the cell membrane, and another sizable proportion devoted to criticizing prevailing conceptions of the nucleus. However, if Brücke wanted to locate the cell’s structure and organization in the protoplasmic “living cell body,” he would have to face the fact that botanists, who had identified protoplasm as the “living substance,” had also defined that substance as a structureless, granular, viscous fluid (Geison 1969; Liu 2017).

To “prove” that protoplasm had material organization, Brücke drew on his expertise in microscopy and vision. In the opening section of “The Elementary Organisms” Brücke compared the problem of microscopic vision to the childhood experience of looking at a jellyfish on the beach. (While most people in the 19th century never saw the beach, Brücke spent his childhood in a place that is famous for its sandy beaches.) The jellyfish is obviously a living organism, but the eye can perceive no organization in the “plate-shaped gelatinous glob with some equally gelatinous appendages.” The problem lies in the object’s composition: the parts that make up its organized structure all transmit and bend light in the same way, *i.e.*, they have the same refractive index. In other words, if the parts of a complex, heterogenous object all have the same or nearly the same index of refraction, then the whole will appear simple and homogenous. Brücke noted that biologists were well acquainted with using histological stains to alter the refractive index of transparent biological tissues.^8^ Brücke argued that staining was not just a practice that added a color, but that, translated into physical or optical terminology, changed the refractive index of the parts that absorbed the stain. This meant that, while a pure empiricist might claim that a jellyfish or protoplasm looked homogenous, the experienced physiologist would know that a complex, organized structure might be hidden behind the microscopist’s *perception* of homogeneity.

**Fig. 1:**
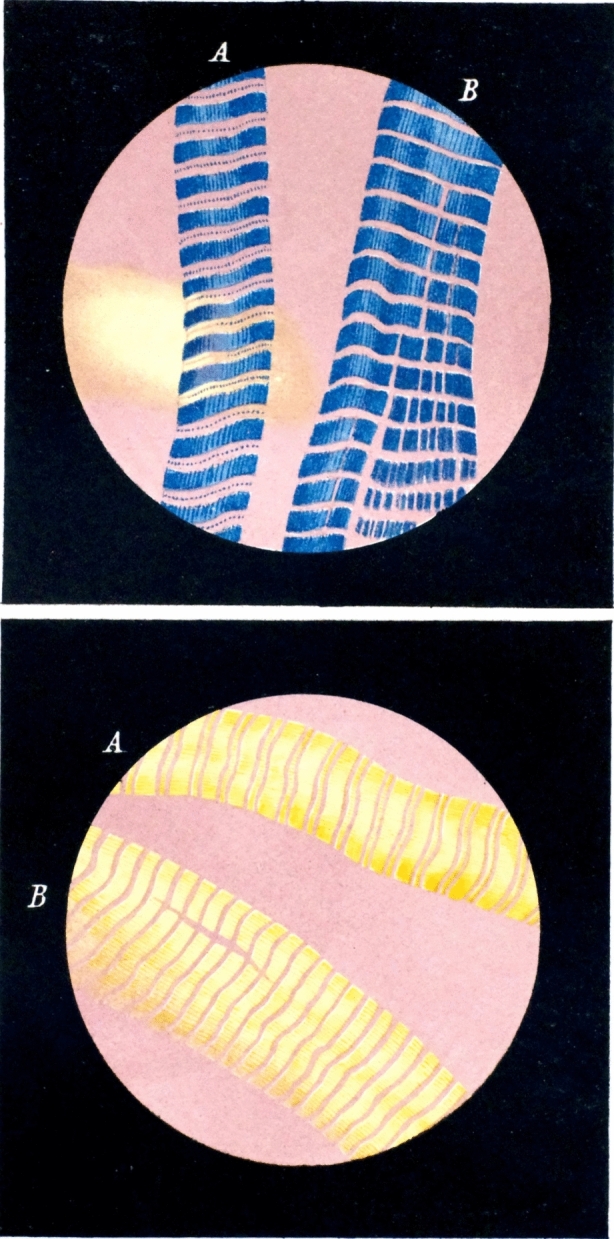
Ernst Brücke’s (1858) illustrations of muscle fibers viewed with polarized light microscopy. The upper and lower views are of the same specimen, but rotated 90°, showing the apparent change in color from blue to yellow of the birefringent disdiaclasts under polarized light.

Brücke had considerable expertise in showing how complex cells could be. One of his first projects upon arriving in Vienna in 1849 was to explore the possibilities of modifying the microscope for use with polarized light.^9^ Polarized light microscopy (PLM) was a relatively new technique (Mohl 1858), and one of the earliest explorations of biological materials with PLM had been made for the Berlin Physical Society by Karl von Erlach (1847). Briefly, the technique required inserting two light polarizing prisms into the microscope, one below the stage and the second between the objective lens and the eyepiece.^10^Under a polarization microscope, a microscopist can see if a specimen that ordinarily appears clear, homogenous, or structureless actually has multiple elements—each with different optical properties, and each appearing in different colors depending on the specimen’s angle relative to the two polarizing prisms. It can also detect materials that are birefringent, *i.e.*, if the specimen has parts that reorient light waves that pass through it, in addition to refracting them. In 1858 Brücke showed that muscle fibers looked much more complex when viewed with PLM than if they were viewed using an ordinary light microscope: they had regular zones of fine, anisotropic bodies arranged parallel to the axis of the muscle fiber, and which, when viewed through polarized light, stood out colorfully against an isotropic (transparent) ground substance (Fig. 1; Brücke 1858). Brücke named the birefringent zones of the muscle fiber “sarcous elements,” and proposed that they were composed of fibrillar or rod-shaped “disdiaclasts”—what we would today call the A-band of myosin fibers of the sarcomere (Galler 2015; Kuhtz-Buschbeck et al. 2021).

Given his commitment to a concept like “organization,” it is perhaps odd to think of Brücke as a stringently physicalist, anti-vitalist biologist. However, Brücke held a nuanced view of the physical complexity of living organisms that he had learned as Müller’s student. He was convinced that “structureless,” fluid protoplasm also possessed a complex organization whose structure was only hidden from view (Rheinberger 2000). Whether he could convince anyone else of this was another matter.

## The Composition and Reception of “The Elementary Organisms”

**Fig. 2:**
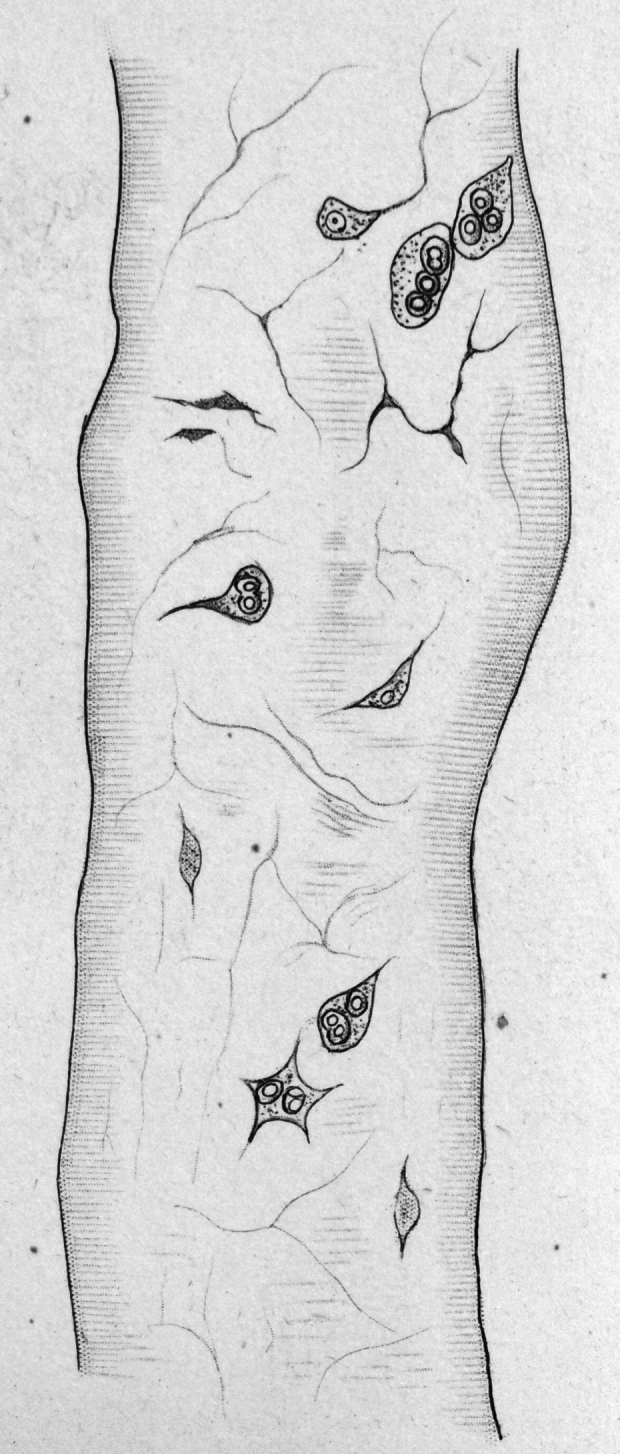
A later illustration by Wilhelm Waldeyer (1865) of a frog muscle fiber, showing the spindle shaped *Muskelkörperchen* with one or more nuclei

Why did Brücke write “The Elementary Organisms” in 1861? From the essay itself we can tell that Brücke had been chafing under the “schema” of the cell as a nucleated, membrane-bound vesicle in the late 1850s, both in his own research and that of his students. “The Elementary Organisms” opens with a long description of work by two of Brücke’s students, Josef Brettauer and Simon Steinach, on the structure of intestinal epithelial cells. According to Brücke, Brettauer, and Steinach, these cells swell osmotically in water only where the cell membrane is the typical thin layer, in the part of the cell facing away from the intestinal lumen. However, the epithelial cells’ microvilli did not swell like a balloon along with the rest of the membrane. Brücke argued they were therefore a distinct structure and not part of the membrane, and further that the membrane of these epithelial cells did not fully surround the cell, but rather formed an open-ended, conical pouch. He argued instead that the microvilli were prismatic extensions of the cell’s protoplasm, and that these cells could not be considered to be fully enclosed by a membrane. (Ironically, today we consider the microvilli as highly folded membranes, held together by proteins.) Brücke also discussed work by his student Tivadar Margó (1816–1896) on muscle structure and development. Margó had been exploring what we now call the muscle satellite cells or muscle stem cells, but which in the 1850s had been described as spots, rings, and cavities, because it was not clear if they were, in fact cells (Fig. 2; Scharner and Zammit 2011). Hermann Welcker (1857) and Brücke’s students called these *Muskelkörperchen* (“muscle corpuscles”), a controversial name that was meant to draw an analogy to Rudolf Virchow’s (1851) general theory of connective tissue morphology (Anderson 1986). Margó argued that maturing *Muskelkörperchen* differentiated into a homogenous ground substance and highly refractive, yellow granules: he proposed that the refractive granules in the *Muskelkörperchen* were the precursors to Brücke’s birefringent disdiaclasts, and that groups of *Muskelkörperchen* fuse together to form mature muscle fibers. While Margó found it easy to identify nuclei in the *Muskelkörperchen*, he wrote that it was “extremely difficult to convince oneself of the existence of a real cell membrane.” Furthermore, under the usual definition of the cell as a membrane-bound vesicle, Margó needed some hypothesis about what happened to this membrane when the *Muskelkörperchen* fused together, even though he could hardly see it. He had to reconcile himself to the notion that it was “very likely that the cell membrane and the contractile contents grow intimately together,” making the membrane impossible to distinguish (Margo 1859, p. 223).

**Fig. 3:**
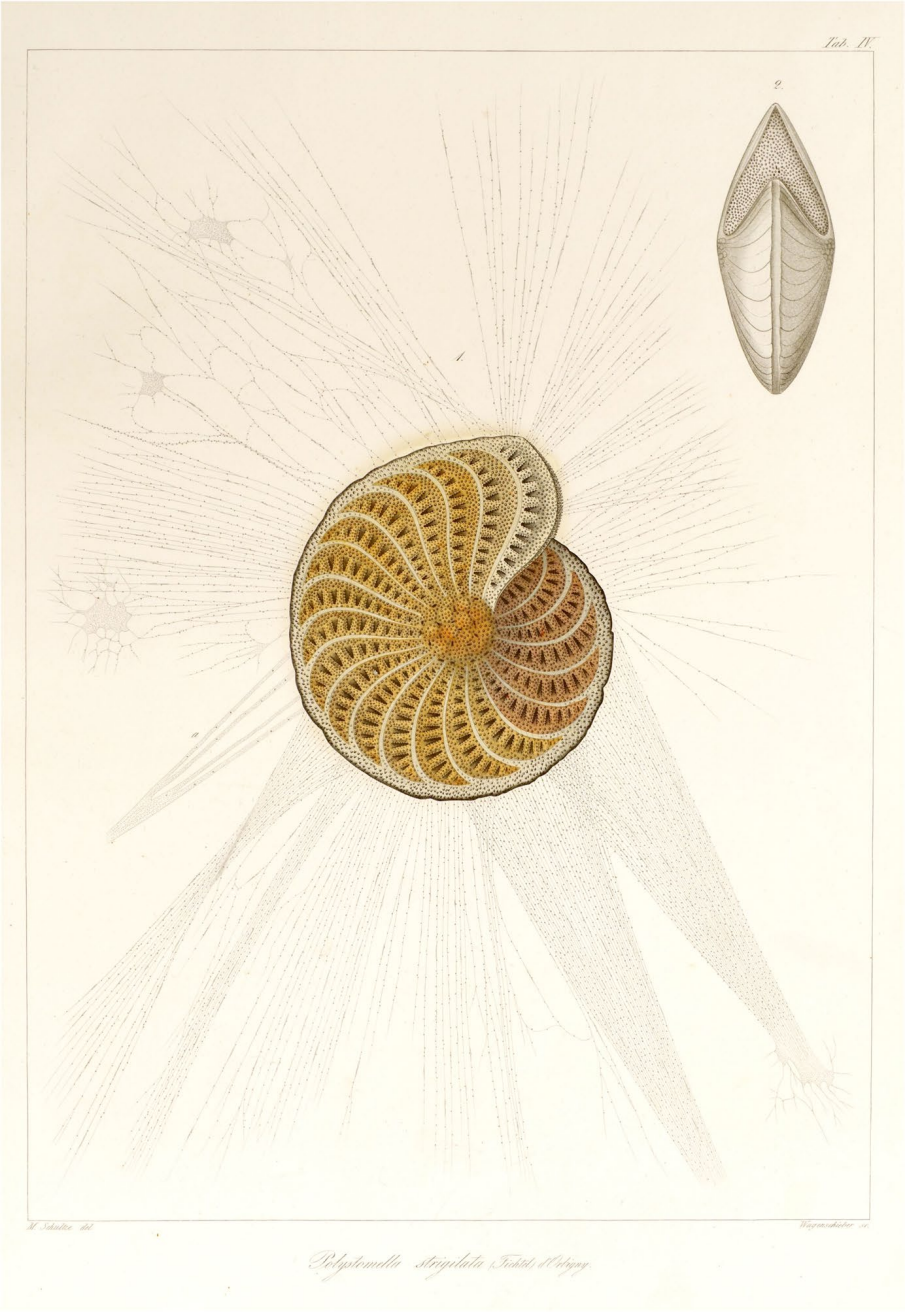
Max Schultze’s (1854) illustration of the testate amoeba *Polystomella strigilata* (Fichtel) d’Orbigny, with outstretched, protoplasmic pseudopoda. Schultze noted that he illustrated the test at 72× magnification, but the pseudopoda at 300× to emphasize their fineness, delicacy, and lack of internal structure

Brücke did not cite Margó’s 1859 study of the *Muskelkörperchen*, likely because it had just had a prominent role a few months earlier in an essay written by Max Schultze, “On *Muskelkörperchen* and That Which is Called a Cell” (Schultze 1861). Schultze’s paper was published in the *Archiv für Anatomie, Physiologie und wissenschaftliche Medicin,* the Berlin-based journal co-edited by the anatomist Carl Bogislaus Reichert (1811–1883) and Brücke’s old friend Emil du Bois-Reymond. It is not inconceivable that du Bois-Reymond tipped Brücke off about Schultze’s essay, and it is also possible that Brücke and Schultze were already acquainted with each other. Schultze had grown up and studied medicine in Greifswald, 35 km southeast from Brücke’s hometown of Stralsund. In the winter semester of 1846–1847 Schultze went to Berlin, attending Brücke’s lectures on the uses and theory of the microscope and Müller’s lectures on comparative anatomy (Schwalbe 1874). Schultze spent much of the 1850s studying protozoa, in particular the foraminifera, a large class of testate amoeba (Fig. 3). In the 1850s most biologists still did not consider such protozoa to be cellular (Churchill 1989; Jacobs 1989; Richmond 1989; Rothschild 1989), at the same time as Schwann’s cell theory left it unclear if cells as “elementary parts” existed in organisms so tiny that their organization was invisible. After reading Margó’s paper and his struggle to show the existence of a cell membrane in the *Muskelkörperchen*, Schultze seized an opportunity to initiate a larger change to the cell concept, one which could also settle the question of whether protozoa were cells.

Schultze reframed the larger debate around the nature of *Muskelkörperchen* as a problem that could be solved if biologists first allowed formless, shape-shifting amoeba to be cells. First, Schultze argued that the amoeba he studied had not been considered cellular because they lacked a clear membranous boundary, just like Margó’s *Muskelkörperchen*. Second, Schultze reminded his fellow animal anatomists that, 10 years previous, the botanist Ferdinand Cohn (1828–1898) had made an analogy between the contractile protoplasm in plant cells and contractile bodies of amoebas: Cohn had defined a plant cell as protoplasm bound within a rigid cell wall, and an animal cell as unbound protoplasm (Cohn 1850, p. 664; Fauré-Fremiet 1935; Geison 1969; Churchill 1989; Klemm 2003; Liu 2017). Borrowing from Cohn, Schultze argued that the membrane-enclosed cell was merely protoplasm in a confined state: “A cell with a membrane that is chemically different from the protoplasm is like an encysted infusorium, like an imprisoned monster [*Ungethüm*]” (Schultze 1861, p. 21). It would be just as easy, Schultze argued, for zoologists to conceptualize the animal egg cell and embryo cells—“the most important cells”—as protoplasm trapped inside a protective skin or shell from which the newborn animal must escape (Schultze 1861, p. 8). This idea of the cell as a living, “more or less fluid body” that secretes a passive shell had been ventured before by the botanist Alexander Braun (1851, pp. 165–166), and it would be echoed by Brücke as well. With the membrane relegated to a secondary byproduct of the cell’s life, Schultze offered a new, membraneless definition for the cell: “A cell is a glob of protoplasm in whose interior lies a nucleus” (*Eine Zelle ist ein Klümpchen Protoplasma, in dessen Innerem ein Kern liegt*; Schultze 1861, p. 11). Schultze not only borrowed the term “protoplasm” from botany, but he also borrowed botanists’ description of protoplasm as “hyaline,” a “viscous, slimy mass filled with granules,” and a substance “held together by its own consistency” (Schultze 1861, pp. 9–10, 16). And Schultze would have felt comfortable with this emphasis on protoplasm’s fluidity, viscosity, and apparent lack of internal structure: the animals he studied were fluid and protean, and created intricate structures only as external shells.

Schultze’s essay “On *Muskelkörperchen*” had a greater impact on biological thought writ large compared to Brücke’s “The Elementary Organisms,” because Schultze successfully made the amoeba into the archetype of the cell. This was a significant change: traditionally the idea of the cell had been grounded in studies of higher plants, going back to the seventeenth century works by Robert Hooke, Nehemiah Grew, and Marcello Malphigi (Harris 1999), where the terms “cell,” “vesicle,” “utricle,” and “saccule” all connoted rows of chambers made of featureless walls. Hooke’s term “cell” and its equivalents across European languages had maintained a living metaphorical link between the structure of organisms and the architectural layout of basement storage rooms, chambers, and monastic cells—and not their contents or inhabitants.^11^ It cannot be underestimated how radical it was for Schultze, Brücke, and their counterparts in botany to suggest that solid walls and membranes were secondary structures secreted by what they believed to be the true seat of cellular life, protoplasm. The very word “cell” had connoted walled chambers for centuries, and Schultze and Brücke sought nothing less than to kill the metaphor (Reynolds 2022). But whereas Brücke’s “The Elementary Organisms” devoted most of its energy to attacking older arguments for the necessity of the cell membrane, Schultze held up the amoeba as a positive example for biologists to study as the exemplary cell (Reynolds 2008). By the end of the 19th century Schultze’s amoeboid conception of the cell would hold a commanding place in popular science as well as in evolutionary theory, promoted by Ernst Haeckel, T. H. Huxley, Claude Bernard, C. S. Peirce, and to a lesser extent Max Verworn (Geison 1969; Brain 2015; Pearce 2018; Bolduc and Angleraux 2023).

In 1861, however, few would have anticipated that unicellular organisms would become so important to the definition of the cell, because most biologists’ direct experience with cells remained within animal histology and botany. Whereas Schultze’s 1861 essay had approached the *Muskelkörperchen* and cell theory from the perspective of protozoa—animals that had never found their place in cell theory—Brücke’s 1861 essay reflected years of frustrations over the place of cell theory in his own physiological research on the eye, muscle, intestines, etc. It is plausible that Brücke was prompted to write “The Elementary Organisms” precisely to emphasize the importance of Schultze’s ideas to histology and physiology in higher animals, and to show that cell theory “from above,” as it were, presented challenges that protozoa did not. Besides, Schultze’s paper had dealt with muscle structure and cited Brücke and Margó, as well as a third student of Brücke’s, Alexander Rollett: given his prominence in muscle physiology Brücke could hardly ignore Schultze’s “On *Muskelkörperchen*,” even though Schultze was an outsider to that area of research.

Brücke disagreed with Schultze’s redefinition of the cell in only two points, albeit significant ones. Brücke did not believe the nucleus was a necessary part of cells, and he did not believe it was adequate to describe protoplasm as a viscous slime. Brücke’s opinion on the nucleus is the most quixotic part of “The Elementary Organisms”: it was probably more of a reckless attack on Schwann’s nuclear-centric theory of cell genesis than a reasoned reconceptualization of the cell. However, Brücke’s second critique—that protoplasm could not be a structureless fluid—came from his training in the Müllerian theories of the organism, and pitted him against Schultze and some botanists’ recent theories of the cell and protoplasm. Brücke had spent his whole career unraveling the complex structure of cells in higher animals, and his recent work with PLM had reinforced his belief that all tissues were highly organized, even if they did not appear to be at first glance. Indeed, throughout “The Elementary Organisms” Brücke used the term “cell body” (*Zellenleib*), a term he coined to connote bodily organization in a way that Schultze’s description of the cell as a “glob” of protoplasm did not. Thus, while Brücke and Schultze shared similar goals in reforming animal cell theory and bringing it in line with botanists’ cell theories, they had different views on what they thought the cell and protoplasm were. For Schultze the cell was structureless, amoeboid protoplasm with a nucleus; for Brücke, the cell and protoplasm were both organized bodies with definite, albeit hypothetical structure.

Brücke composed “The Elementary Organisms” in the summer of 1861 while vacationing in the Austrian *Salzkammergut*, a popular retreat for Vienna’s elite and intelligentsia (Coen 2007). He was aware that his attack on the most celebrated theory in histology might be too radical for some biologists. He was particularly interested to hear the reaction of a former colleague from his Berlin years, Carl Bogislaus Reichert, yet another of Johannes Müller’s students. After Müller’s death in 1858 his professorship at the University of Berlin was split in two, with Reichert taking the chair of anatomy and du Bois-Reymond taking the chair of physiology. Both Brücke and du Bois-Reymond were eager combatants in the scientific rivalry between physicalist physiology and descriptive anatomy (Rothschuh 1974; Lenoir 1988; Nyhart 1995), a rivalry that informed Brücke’s style in “The Elementary Organisms” as well as its immediate reception. As Brücke wrote to du Bois-Reymond in November 1861:

I brought a microscope and books with me to Unterach, where I hammered out an essay and gave it the title “The Elementary Organisms.” This is what I am calling the cells, and I’ve done it in a way that Father Reichert is probably not going to agree with. I am all the sorrier that he missed [visiting] me here, I could have at least prepared him for the desecration of the temple.^12^

“Father Reichert” was only seven years older than Brücke and du Bois-Reymond, but his teleological ideas about organismal development made him an object of fun for the two physiologists, who admired his skills but thought him hopelessly old fashioned. Brücke’s “abomination” prompted Reichert to write a sixty-five page rebuttal, essentially accusing Brücke of ignoring the laws of inductive reasoning and for trading a known theory of organization—cells with membranes and nuclei—for a hypothetical notion of protoplasm’s as-yet invisible structure (Reichert 1863; see Lenoir 1983, pp. 215–228). Robert Remak joined Reichert’s critique, dismissing the need for any reform to cell theory, let alone on the basis of atypical biological objects: for example, Remak argued that the *Muskelkörperchen* were not cells but rather pathological entities, and that membranes could be revealed by hardening them with chemical reagents (Remak 1862, pp. 241–242; see Anderson 1986). For his part, du Bois-Reymond thought Reichert’s sputtering response deserved some kind of rejoinder, to which Brücke replied:

Dear Emil!

My thanks for both of your recent letters (and best wishes to [Heinrich] Dove). I really had fun with the last one, and it shows me that the old humor hasn’t yet gone to ground. But what are you thinking? I should really start a polemic with Father Reichert! With Father Reichert, who is a living piece of our memories of Berlin anatomy, and thereby an extremely unfit object for polemics. Remember that, twenty years ago, his thinking was not the lowest object of our mutual amusement. He may have said of me that I eat tallow candles for breakfast and dip window shutters in my coffee, but he hasn’t destroyed the feelings of regret I have for him because he has to watch the silent, crumbling decay of everything he crammed into his brain for so many years. The vesicularists [*Bläschenmänner*] cause me little pain. “It’s over, Lady Mary, and you’ll seduce me no more!”^13^

The “vesicularists” (those who insisted the cell was a membrane-bound vesicle) would, indeed, lose out to the reforms proposed by Schultze and Brücke: for the next fifty years, definitions of the cell would focus on protoplasm as the cell’s living body, leaving the nucleus in a state of limbo and the existence of the cell wall or membrane a matter of dispute.

### A Note on Sources

Ernst Brücke is still not a familiar figure for historians, despite the fact that he was among the most highly regarded physiologists of his time. Nevertheless, the sources and other biographical traces are numerous and diverse, and students looking for projects in the history of physiology may find Brücke a useful starting point. The chief published sources of information on Brücke’s life and science are, (1) the biography written in 1928 by his grandson, Ernst Theodor von Brücke, which includes a complete list of Brücke’s publications; (2) Erna Lesky’s magisterial book, *The Vienna Medical School of the 19th Century* (1965; English translation 1976); (3) the collection of letters sent by Brücke to Emil du Bois-Reymond, compiled, annotated, and published by his great-grandson Hans Brücke in 1978; and (4) a collection of letters from Brücke to Hermann Helmholtz, compiled and published in 1994 by Herbert Hörz and Marie-Luise Körner. Broad context for Brücke’s work and life can be gleaned from Laura Otis’ group biography of Johannes Müller and his students, *Müller’s Lab* (2007), Gabriel Finkelstein’s biography of Emil du Bois-Reymond (2013), and from the sizable scholarship on Hermann Helmholtz (e.g., Cahan 1993; Hörz and Körner 1994; Cahan 2018; Wise 2018). An unexpected source of information on Brücke is the scholarship on Sigmund Freud, who studied under Brücke from 1876 to 1882; this includes Freud’s correspondence (e.g., in Freud 1960; Boehlich 1990), and some scholarship that veers off into psychoanalytical fantasies (Cranefield 1966b; Rosenkötter 1971; Rosen 1972; Widder 1999; Yılmaz 2022).

## Footnotes

1 Letter from Emil du Bois-Reymond to Eduard Hallmann, Berlin, May 1842, in du Bois-Reymond (1918, p. 108).

2 See the letters from Ernst Brücke to Emil du Bois-Reymond, Vienna, 21 Dec 1856 (No. 78) and 15 Nov 1867 (No. 152), respectively, in Brücke et al. (1978).

3 Du Bois-Reymond told their mutual friend Carl Ludwig that on Saturday, March 18, the day of the major outbreak of violence, Brücke had gone out to see the “extensive riot” in front of the Royal Palace, but that Brücke, “unarmed and coming up against the military from behind wherever he went, he had no other choice but to stay in his apartment.” Letter from Emil du Bois-Reymond to Carl Ludwig, Berlin, 22 Apr 1848, in du Bois-Reymond et al. (1982, p. 10). Brücke’s address at the time was at Dorotheenstraße 2; the Palace would have been in full view if Brücke had left his apartment and walked twenty paces to his right, and from there the site of the demonstration at Schloßplatz would be a six minute walk away. Brücke’s apartment was also across the street from the home and private laboratory of physicist Gustav Magnus, who hosted the Berlin Physical Society’s meetings (at Am Kupfergraben 7; see Wise 2018, chap. 6), and halfway between the university and Johannes Müller’s house (Cantianstraße 7, at what is today the northeastern side of the Pergamon Museum).

4 Letter from Brücke to du Bois-Reymond, Königsberg, 22 Feb 1849 (No. 19), in Brücke et al. (1978).

5 Müller wrote this short preface for the second edition of his *Handbuch der Physiologie des Menschen* (1834), where he clarified his use of the terms “life,” “organization,” and “organic,” as well to address reviewers’ concerns about his claims about the nature of blood.

6 Schwann famously compared cell genesis to crystallization in minerals, a comparison that led many historians to rank him among the mechanists (e.g., Maulitz 1971; Rothschuh 1974). However, Florence Vienne (2017) has convincingly argued that this comparison needs to be understood in light of his Catholicism: Schwann privileged God as the ultimate cause of organization, and rejected Müller’s theory of vital forces as depriving God of that ultimate agency.

7 In medical histology, endogenous free cell formation persisted into the 1860s as a kind of pathological cell genesis (Anderson 1986; Harris 1999). Free cell formation persisted in botany until the 1870s as the special kind of cell genesis for reproductive cells (Farley 1982).

8 Brücke’s student Salomon Stricker (1870, p. *xxxiii*) listed the following staining agents in his histology textbook: carmine, gold chloride, silver nitrate, saffron, indigo carmine, haematoxylin, picric acid, palladium chloride, osmic acid, and aniline.

9 Brücke had to order Nicol prisms and a modified microscope from Schiek in Berlin, even though Plössl in Vienna was regarded as making superior microscopes: “Write and tell me if you can get me a pair of Nicols for a microscope from Böttcher and how long it will take Schiek to get one of his 80 Thaler microscopes. Plössl is really as stubborn as an ox.” Letter from Brücke to du Bois-Reymond, Vienna, 12 Oct 1849 (No. 25), in Brücke et al. (1978).

10 The Nicol prism, invented in 1828 by the Scottish geologist William Nicol is a polarizer made from a rhombus of clear calcite crystal, cut at an angle and glued back together with Canada balsam. In the middle of the 20th century these prism polarizers in polarized light microscopes were replaced by thin plastic Polaroid filters, after the latter were invented by Edwin Land in the 1930s.

11 Or, to quote Robert Hooke (1665, p. 113), that cells consist of “a very small quantity of a solid body, extended into exceedingly large dimensions.” Compare the historical entries for “cell” in the *Oxford English Dictionary* and *Zelle* in the *Deutsches Wörterbuch von Jacob und Wilhelm Grimm* (vol. 31, 1956, pp. 603–609). I agree with Winifred Peters (2023) that Hooke did not intend his use of the word “cell” to invariably mean *sealed* chambers, and that it is worth further investigation as to when in the 19th century this “dogmatic” reading of Hooke entered the historiography.

12 Letter from Brücke to du Bois-Reymond, Vienna, 22 Nov 1861 (No. 117), in Brücke et al. (1978). Brücke had invited du Bois-Reymond to vacation together in Unterach, and he may have extended the invitation to Reichert.

13 Letter from Brücke to du Bois-Reymond, Vienna, 24 Jan 1863 (No. 127), in Brücke et al. (1978). Brücke is quoting a line (slightly inaccurately) from Friedrich Schiller’s *Maria Stuart*, act 3, scene 4.
